# Development of Remineralizing and Antibacterial Resin Coating for Provisional Crowns with Improved Bond Strength and Wear Resistance

**DOI:** 10.3390/polym18080945

**Published:** 2026-04-12

**Authors:** Ibrahim Ba-Armah, Abdullah Alhussein, Nader Almutairi, Mohammad Alenizy, Heba Alqarni, Yazeed Altamimi, Ayman Altamimi, Radi Masri, Jirun Sun, Michael D. Weir, Hockin H. K. Xu

**Affiliations:** 1 Dental Biomedical Sciences PhD Program, Graduate School, University of Maryland, Baltimore, MD 21201, USA; 2 Department of Biomaterials and Regenerative Dental Medicine, University of Maryland School of Dentistry, Baltimore, MD 21201, USA; michael.weir@umaryland.edu; 3 Department of Restorative Dental Sciences, College of Dentistry, Imam Abdulrahman Bin Faisal University, Dammam 31441, Saudi Arabia; 4 Department of Restorative Dental Science, College of Dentistry, King Saud University, Riyadh 11545, Saudi Arabia; aalhussein@ksu.edu.sa; 5 Department of Conservative Dental Sciences, College of Dentistry, Prince Sattam bin Abdulaziz University, Alkharj 16245, Saudi Arabia; 6 Department of Restorative Dental Sciences, College of Dentistry, University of Hail, Hail 55475, Saudi Arabia; 7 Department of Pediatric Dentistry and Orthodontics Sciences, College of Dentistry, King Khalid University, Abha 61421, Saudi Arabia; 8 Department of Advanced Oral Sciences and Therapeutics, University of Maryland School of Dentistry, Baltimore, MD 21201, USA; 9 The ADA Forsyth Institute, Cambridge, MA 02142, USA; 10 Center for Stem Cell Biology & Regenerative Medicine, University of Maryland School of Medicine, Baltimore, MD 21201, USA; 11 Marlene and Stewart Greenebaum Cancer Center, University of Maryland School of Medicine, Baltimore, MD 21201, USA

**Keywords:** bioactive dental materials, antibacterial resin, provisional crowns, antibacterial coating, amorphous calcium phosphate nanoparticles, calcium fluoride nanoparticles, remineralization, ion release

## Abstract

Secondary caries and biofilm accumulation remain major causes of failure in provisional crowns and restorations, highlighting the need for multifunctional resin coatings with antibacterial and remineralizing capabilities. This study aimed to develop a novel bioactive and antibacterial resin-based surface coating incorporating 10% dimethylaminododecyl methacrylate (DMADDM), 20% nanoparticles of amorphous calcium phosphate (NACP), and/or 20% calcium fluoride nanoparticles (nCaF_2_) within a urethane dimethacrylate/triethylene glycol divinylbenzyl ether (UDMA/TEG-DVBE) matrix. Coatings were evaluated for degree of conversion (DC), flow, shear bond strength, brushing wear resistance (10,000 cycles), and calcium (Ca), phosphate (PO_4_), and fluoride (F) ion release up to 70 days. All groups achieved clinically acceptable polymerization, with the lowest DC at 50%. NACP-containing coatings significantly increased shear bond strength to 18.3 ± 2.8 MPa, representing a ~170% increase compared with the experimental control (6.8 ± 2.1 MPa) and exceeding the ISO 10477 minimum threshold of 5 MPa. After brushing simulation, experimental coatings demonstrated low wear depth (0.93–1.19 µm), which was ~40% lower than the commercial control (1.85 ± 0.40 µm). Sustained ion release was achieved for 70 days, with 20% NACP-formula releasing 1.22 mmol/L Ca and 0.90 mmol/L PO_4_, while the dual NACP–nCaF_2_ formulation provided simultaneous Ca (0.62 mmol/L) and F (0.33 mmol/L) release. The developed coatings demonstrated promising physicochemical properties, bonding performance, wear resistance, and sustained remineralizing ion release, supporting their potential application as therapeutic surface coatings for provisional restorations.

## 1. Introduction

Dental caries remains one of the most prevalent chronic diseases worldwide and continues to represent a significant global health burden. Secondary caries at restoration margins is a major cause of failure in both provisional and definitive restorations. This has led to repeated interventions and increased costs [1,2,3]. Provisional restorations are often used in fixed prosthodontics to protect prepared teeth, maintain occlusal stability, and preserve periodontal health until a definitive prosthesis is delivered. In specific cases, these materials remain in the oral cavity for extended periods therefore are susceptible to bacterial colonization, surface degradation, and wear [4,5]. The development of advanced dental materials with therapeutic functionality has become a major focus for researchers [6].

Conventional provisional materials are primarily based on polymers formed from urethane dimethacrylate (UDMA) and bisphenol A-glycidyl methacrylate (Bis-GMA) monomers. Throughout history, these systems provided acceptable mechanical and aesthetic properties. However, they are biologically passive and lack the ability to inhibit bacterial growth or promote remineralization [7]. The accumulation of cariogenic biofilms, especially those dominated by *Streptococcus mutans*, results in localized acid production, demineralization of adjacent tooth structure, and increased risk of secondary caries [8,9]. Therefore, the modification of the resin polymer systems to introduce antibacterial and bioactive properties has emerged to enhance restoration longevity.

One of the promising approaches involves the incorporation of polymerizable quaternary ammonium methacrylates (QAMs) into dental resins. The presence of cationic monomers in QAMs allows them to covalently integrate into the polymer network during photopolymerization, providing long-term antibacterial activity through contact-killing mechanisms [10]. Dimethylaminododecyl methacrylate (DMADDM) has demonstrated strong antibacterial efficacy by disrupting bacterial membranes without compromising mechanical properties or biocompatibility [11]. Recent studies have reported that QAM-containing materials can reduce bacterial adhesion, metabolic activity, and lactic acid production in dental biofilms significantly [12]. However, antibacterial activity alone is insufficient to address the mineral loss associated with the caries process.

Tooth demineralization occurs when biofilm accumulation on the tooth surface produces lactic acid, initiating mineral loss and subsequent secondary caries formation. Remineralization involves repairing degraded crystals by depositing calcium and phosphate ions derived from an external source [13]. Fluoride is widely regarded as the cornerstone of non-cavitated caries lesion management; however, it requires calcium and phosphate ions to form fluorapatite or fluorohydroxyapatite [14]. Developing a bioactive resin-based provisional crown coating capable of releasing calcium, phosphate, and fluoride ions could therefore play a pivotal role in promoting tooth remineralization. Calcium phosphate (CaP) filler particles incorporated into resin-based materials have demonstrated the ability to promote hard tissue regeneration and prevent secondary caries [14,15]. During acidic challenges, CaP fillers release Ca^2+^ and PO_4_^3−^ ions, thereby enhancing tooth mineral regeneration and inhibiting further mineral loss [16,17]. Nevertheless, conventional CaP fillers, with particle sizes ranging from 1 to 55 μm, exhibit insufficient mechanical properties and limited ionic release when incorporated into resin-based materials [16,17,18]. To address these limitations, nanoparticles of amorphous calcium phosphate (NACP) have been developed, offering greater surface area and improved mechanical performance [19]. Human in situ studies have demonstrated that incorporating NACP into resin-based materials significantly enhances mineral regeneration in tooth structure compared to commercially available fluoride-releasing restorative materials [20]. Commercial restorative materials with fluoride-releasing capabilities frequently suffer from short-term release profiles or compromised mechanical properties [21,22]. Incorporating calcium fluoride nanoparticles (nCaF_2_) into resin-based materials has emerged as an effective strategy to overcome these limitations, enabling sustained fluoride release without adversely affecting mechanical properties [23]. The combined NACP–nCaF_2_ formulation proposed in the present study offers dual ion release, supporting a synergistic approach to caries prevention. Previous studies have reported that such multifunctional systems have shown considerable promise in simultaneously reducing tooth demineralization and enhancing remineralization, underscoring the clinical rationale for the coating strategy developed in the present study [24,25].

Bioactive resin systems capable of releasing remineralizing ions have gained considerable attention in recent years. These nanoparticles, as shown in previous studies, have been successfully incorporated into resin matrices, enabling sustained ion release and enhanced resistance to demineralization while maintaining good mechanical properties [20,26]. Several nanoscale modifier strategies have been explored for incorporation into resin-based dental materials to impart remineralizing and antibacterial properties. Hydroxyapatite nanoparticles (nHA) have been investigated for their remineralizing potential; however, their relatively low mechanical strength and fracture toughness limit their suitability for load-bearing applications, and their remineralizing effect is largely confined to the surface layer of carious lesions [27]. Silver nanoparticles (NAg) have demonstrated potent antibacterial activity through sustained silver ion release; however, concerns regarding cytotoxicity at higher concentrations, color instability, and the absence of remineralizing capability represent significant limitations for their use as standalone modifiers in provisional restorative applications [27]. Bioactive glass (BG) has been shown to promote hydroxyapatite formation and remineralization; however, its incorporation into resin matrices is associated with progressive deterioration of mechanical properties with increasing filler levels, and its remineralizing surface must remain in direct contact with the oral environment, limiting the degree of hydrophobic matrix modification achievable [28]. Metal oxide nanoparticles such as ZnO and TiO_2_ have also been evaluated for their antibacterial properties in dental resins; however, they lack inherent remineralizing capability and raise biocompatibility concerns at effective antibacterial concentrations [27,28].

In contrast, the components selected for the present study offer distinct and complementary advantages. NACP, synthesized via a spray-drying technique, provides a high surface area and pH-responsive Ca^2+^ and PO_4_^3−^ ion release that increases under cariogenic acidic conditions, enabling targeted remineralization precisely when it is most needed. Importantly, NACP has been shown to maintain mechanical properties comparable to commercial composites when combined with reinforcing glass co-fillers [19,20]. DMADDM was selected as the antibacterial agent due to its ability to covalently integrate into the polymer network via photopolymerization, providing durable contact-killing activity without leaching, and without adversely affecting mechanical properties. These advantages address the key limitations of releasable antibacterial agents such as NAg [29]. Finally, nCaF_2_ was incorporated to provide sustained fluoride release without the short-term release profiles associated with conventional fluoride-releasing materials, enabling the simultaneous delivery of calcium, phosphate, and fluoride ions for synergistic remineralization [23,30]. The combination of these three components within a single resin-based coating therefore represents a rational and evidence-based selection strategy that addresses the specific limitations of existing nanoscale modifier approaches.

The advancement in nanotechnology and polymer engineering has enabled the design of multifunctional materials that combine antibacterial agents and remineralizing fillers [31]. Studies have demonstrated synergistic effects when calcium phosphate and fluoride-releasing systems are incorporated into dental polymers, resulting in improved remineralization and antibacterial performance [32]. Moreover, the nanoscale size of these fillers enhances ion-release kinetics while minimizing adverse effects on the polymer network structure and mechanical properties [33,34].

In addition to the incorporation of antibacterial and bioactive components, the resin matrix plays a significant role in determining the physicochemical stability, durability, and long-term clinical performance of multifunctional coatings. In this study, a polymer system based on UDMA and triethylene glycol divinylbenzyl ether (TEG-DVBE) was employed. UDMA-based resins have been widely used due to their relatively low viscosity, flexible molecular structure, and ability to form highly crosslinked polymer networks with improved toughness and reduced polymerization shrinkage compared with conventional bisphenol A glycidyl methacrylate (bis-GMA) systems [35,36,37]. Furthermore, UDMA exhibits enhanced mechanical strength, improved degree of conversion, and reduced water sorption, which contribute to long-term durability and resistance to hydrolytic degradation [38].

The incorporation of ether-based monomers such as TEG-DVBE provides additional advantages by reducing ester content within the polymer backbone, thereby improving resistance to hydrolysis and enzymatic degradation in the oral environment. In contrast to traditional methacrylate systems, vinyl ether and divinylbenzyl ether monomers exhibit reduced susceptibility to esterase-mediated degradation and may enhance the longevity of resin-based materials. Moreover, TEG-DVBE can improve polymerization kinetics and network homogeneity due to its flexible structure and lower polymerization stress, which are beneficial for maintaining interfacial integrity and minimizing marginal break down [39,40]. Recent studies have also demonstrated that ether-based polymer systems show improved resistance to bacterial and salivary enzymatic activity, making them attractive candidates for long-term restorative and coating applications [41]. Therefore, the combination of UDMA and TEG-DVBE provides a strong and hydrolytically stable polymer matrix that is well suited for the incorporation of antibacterial and remineralizing agents.

Despite these advancements, several challenges remain. The integration of antibacterial monomers and bioactive nanoparticles may affect polymerization behavior, filler dispersion, and bonding durability. In addition, limited research has evaluated the long-term performance of such coatings under simulated oral conditions, including mechanical wear and ion-release stability. Therefore, further investigation is required to optimize the formulation and functional performance of multifunctional resin coatings for provisional dental applications. Developing surface coatings allows introducing a multifunctional system into provisional restorations. Unlike bulk modification, surface coatings permit the functionalization of existing materials without compromising their structural integrity. Multifunctional polymer coatings may improve wear resistance, reduce bacterial adhesion, and provide sustained release of therapeutic ions. These systems also offer clinical advantages, as they can be applied chairside and adapted to various restorative materials [42].

Several multifunctional resin-based systems combining antibacterial and remineralizing agents have been previously reported. Fei et al. developed a pit and fissure sealant incorporating nCaF_2_ and DMAHDM with dual fluoride release and antibacterial functionality, but without calcium phosphate nanoparticles [43]. More recently, Almutairi et al. reported a multifunctional root surface coating incorporating NACP and DMAHDM within the same UDMA/TEG-DVBE matrix used in the present study, but without nCaF_2_ and therefore without simultaneous triple ion release [44]. To the best of our knowledge, no prior study has developed a provisional crown surface coating combining DMADDM, NACP, and nCaF_2_ within a UDMA/TEG-DVBE matrix with a comprehensive evaluation of physicochemical properties, bond strength, wear resistance, and sustained calcium, phosphate, and fluoride ion release.

Building upon our previously published work establishing the antibacterial efficacy and biocompatibility of DMADDM-modified UDMA/TEG-DVBE resin coatings for provisional crowns [42], the present study extends this research by incorporating remineralizing bioactive nanoparticles to develop a multifunctional coating with combined antibacterial and remineralizing capabilities. Thus, the objective of the present study was to develop and characterize a novel antibacterial and remineralizing resin-based surface coating incorporating DMADDM, nanoparticles of amorphous calcium phosphate, and calcium fluoride nanoparticles within a UDMA/TEG-DVBE-based polymer system. The physicochemical properties, bonding performance, wear resistance, and sustained release of calcium, phosphate, and fluoride ions were evaluated. The hypotheses tested in this study are as follows:

1. The incorporation of antibacterial and bioactive components would not adversely affect the polymerization and mechanical performance of the experimental formulations.
2. The experimental formulations would demonstrate increased wear resistance compared to the commercial control.
3. The experimental formulations would demonstrate sustained release of calcium, phosphate, and fluoride ions.

## 2. Materials and Methods

### 2.1. Synthesis of Novel Bioactive Resin-Based Coatings

Resin-based coating was synthesized using a 55.8 wt% mass fraction of urethane dimethacrylate (UDMA; Esstech, Essington, PA, USA) and a 44.2 wt% mass fraction of triethylene glycol divinylbenzyl ether (TEG-DVBE) at a 1:1 molar ratio [41]. Triethylene glycol was reacted with sodium hydride in dimethylformamide (DMF) under an argon atmosphere at 4° temperature. Subsequently, 4-vinylbenzyl chloride was added dropwise, and the reaction was allowed to proceed at room temperature. The mixture was then quenched with saturated ammonium chloride, followed by aqueous dilution and extraction with ethyl acetate. The organic phase was washed, dried, and concentrated. The crude product was purified by flash column chromatography to obtain TEG-DVBE as a pale-yellow oil, as described in a previous study [39]. Camphorquinone (CQ; Millipore Sigma, St. Louis, MO, USA) was incorporated at 0.2 wt% as a photoinitiator, and ethyl 4-N,N-dimethylaminobenzoate (4EDMAB; Millipore Sigma, St. Louis, MO, USA) was added at 0.8 wt% as a co-initiator. Throughout this study, this formulation is referred to as the UV resin.

The antibacterial quaternary ammonium monomer dimethylaminododecyl methacrylate (DMADDM) was synthesized via a modified Menschutkin reaction by combining tertiary amines with organo-halides. Specifically, 10 mmol of 1-bromododecane (BDD; TCI America, Portland, OR, USA) and 10 mmol of 2-(dimethylamino)ethyl methacrylate (DMAEMA; Millipore Sigma, St. Louis, MO, USA) were dissolved in 3 g of ethanol and reacted in a sealed glass container at 70 °C for 24 h. Following solvent evaporation, DMADDM was obtained as a waxy white solid [45].

Nanoparticles of amorphous calcium phosphate (NACP) were prepared using a spray-drying method, as previously described [19]. Calcium carbonate and dicalcium phosphate anhydrous were dissolved in acetic acid to obtain final concentrations of 8 mmol/L calcium and 5.333 mmol/L phosphate, resulting in a Ca/P molar ratio of 1.5, corresponding to that of amorphous calcium phosphate [Ca_3_(PO_4_)_2_]. The solution was spray-dried in a heated chamber, and the particles were collected using an electrostatic precipitator. NACP was incorporated to enable sustained release of calcium and phosphate ions to promote remineralization while maintaining mechanical integrity.

Nano-sized calcium fluoride (nCaF_2_) particles were also produced using a spray-drying technique [46]. A calcium hydroxide solution (2 mmol/L) and an ammonium fluoride solution (4 mmol/L) were simultaneously atomized into a heated chamber at a flow rate of approximately 10 mL/min. The resulting nanoparticles were collected using an electrostatic precipitator. These fillers were incorporated to provide calcium and fluoride ion release for remineralization without compromising mechanical properties.

Four experimental resin coatings were formulated based on the UV resin system. The experimental control resin (Experimental control) consisted of UV resin containing 20 wt% silanized barium boroaluminosilicate glass particles (median size: 1.4 μm; Dentsply Sirona, Milford, DE, USA) as reinforcing fillers. The second formulation (10DMADDM + 20NACP) contained 10 wt% DMADDM and 20 wt% NACP as nano-fillers. The third formulation (10DMADDM + 20nCaF_2_) consisted of UV resin containing 10 wt% DMADDM and 20 wt% nCaF_2_. The fourth formulation (10DMADDM + 10NACP + 10nCaF_2_) included 10 wt% DMADDM and 20 wt% total nano-fillers composed of 10 wt% NACP and 10 wt% nCaF_2_.

The experimental resin coatings were formulated by incorporating the corresponding filler contents into the UV resin using a rapid mixer (SpeedMixer™ DAC 150.1 FVZ-K, Hauschild Inc., Farmington Hills, MI, USA) for 60 s at 2500 rpm.

A commercially available surface sealant, OptiGuard^®^ (Kerr Dental, Brea, CA, USA), was used as a commercial control and is referred to as (Opti control). This material is a light-cured, fluoride-releasing, unfilled resin primarily based on triethylene glycol dimethacrylate (TEGDMA). In addition, TEMPSMART^®^ provisional crown material (GC America, Alsip, IL, USA) was used as an additional control and is referred to as (Temp control). This material is a dual-cured bis-acrylic composite mainly composed of urethane dimethacrylate (UDMA) and butylated hydroxytoluene (BHT).

The resin groups tested in this study are:

1. TEMPSMART^®^ by GC America, as commercial control (Temp control);
2. OptiGuard^®^ Surface Sealant by Kerr, as commercial control (Opti control);
3. UV resin + 0% DMADDM + 20% glass fillers as Experimental Control (Experimental control);
4. UV resin + 10% DMADDM + 20% NACP as (10DMADDM + 20NACP);
5. UV resin + 10% DMADDM + 20% nCaF_2_ as (10DMADDM + 20nCaF_2_);
6. UV resin + 10% DMADDM + 10% NACP + 10% nCaF_2_ as (10DMADDM + 10NACP + 10nCaF_2_).

Sample sizes for each test were selected based on previously published studies employing equivalent testing protocols. These sample sizes have been demonstrated to provide sufficient statistical power to detect clinically meaningful differences between experimental groups in prior studies using comparable materials and methodologies.

### 2.2. Transmission Electron Microscopy (TEM) of Nanoparticles

The particle sizes of NACP and nCaF_2_ were characterized using transmission electron microscopy (TEM) (Tecnai T12; FEI Company, Hillsboro, OR, USA) operated at an accelerating voltage of 80 kV and a magnification range of 80,000× to 100,000×. To minimize particle agglomeration, the powders were dispersed in ethanol and ultrasonicated prior to analysis. A drop of the suspension was deposited onto carbon-coated copper grids and allowed to dry before imaging. The particle size of the spray-dried NACP and nCaF_2_ powders was determined by analyzing TEM micrographs using ImageJ software (ImageJ.JS version 0.6.0, National Institutes of Health, Bethesda, MD, USA). A total of 50 particles (*n* = 50) were measured for each nanopowder.

### 2.3. Degree of Conversion

The degree of conversion (DC) was determined for all groups using a Fourier transform infrared (FT-IR) spectrophotometer (Nexus 670, Thermo Scientific, Madison, WI, USA) equipped with an infrared source, a mercury cadmium telluride (MCT-A) detector, a potassium bromide (KBr) beam splitter, and an attenuated total reflectance (ATR) accessory (Golden Gate, Specac, Fort Washington, PA, USA). Mid-infrared spectra were obtained by co-adding 32 scans at a spectral resolution of 4 cm^−1^.

A small amount of uncured resin coating (*n* = 3) was placed directly onto the ATR crystal and covered with a Mylar strip. Spectra were collected prior to polymerization. The specimens were then light-cured for 60 s using a light-curing unit (Bluephase Style, Ivoclar Vivadent, Amherst, NY, USA) with a minimum irradiance of 1100 mW/cm^2^, which was verified using a calibrated radiometer (CURE RITE^®^ Visible Curing Light Meter, DENTSPLY Caulk, Milford, DE, USA). Spectra were recorded immediately after light curing.

The aromatic C=C absorption band at 1583 cm^−1^ was used as the internal standard, while the reduction in the aliphatic vinyl methacrylate C=C absorption band at 1637 cm^−1^ was monitored to calculate the degree of conversion. The DC (%) was calculated using the following equation:

$$DC(\%)=1\;-=\frac{A^{\prime }vinyl/A^{\prime }aromatic}{A^{0}vinyl/A^{0}aromatic}\;\times 100$$

where the vinyl methacrylate ($A^{0}\;vinyl)$ and ($A^{0}$ aromatic) aromatic internal standard absorptions are represented before ($A^{0}$) and after (A′) cure [47,48].

### 2.4. Coating Flowability

The flow test was conducted in accordance with ISO 6876:2012 [49]. A volume of 0.05 mL of resin coating from each group (*n* = 3) was dispensed onto a glass plate measuring 100 × 100 × 3 mm. A second glass plate (50 g) was placed on top of the resin, and an additional load of 100 g was applied to the center of the upper plate, resulting in a total applied weight of 150 g. After 10 min, the maximum and minimum diameters of the material flow were measured in millimeters (mm) using a digital caliper. When the difference between the two diameters was less than 1 mm, their mean value was recorded.

### 2.5. Coating Shear Bond Testing

Fifty disc-shaped specimens were produced from TEMPSMART provisional crown material, each measuring 8 mm in diameter and 1 mm in thickness. Polymerization was performed for 90 s on each surface using a laboratory light-curing unit (Labolight DUO, GC America, Alsip, IL, USA). A transparent matrix strip was placed over the material during curing to minimize the formation of an oxygen inhibition layer, and the specimens were compressed between two microscope slides to achieve smooth, flat surfaces The discs were subsequently embedded in self-curing acrylic resin (Lang Dental Manufacturing, Wheeling, IL, USA) using cylindrical metal mold to maintain positional stability and alignment during mechanical testing.

For each of the five resin coating groups, ten specimens were bonded to the prepared disc surfaces using a bonding clamp fitted with a mold insert (Model Nos. 34,224 and 34,228; Ultradent, South Jordan, UT, USA). The bonded assemblies were subsequently light-activated for 60 s using a visible light-curing unit (Optilux VCL 401, Demetron Kerr, Danbury, CT, USA). All specimens were stored in distilled water at 37 °C for 24 h prior to testing. Shear bond strength evaluation was carried out with a Universal Testing Machine (Insight 1, MTS, Cary, NC, USA) equipped with a chisel-type loading blade. A compressive force was applied at a crosshead speed of 0.5 mm/min, with a maximum load capacity of 1 kN, until interfacial failure occurred. Shear bond strength values (MPa) were determined by dividing the maximum load at debonding (N) by the bonded surface area (mm^2^). The experimental procedure was conducted in accordance with previously established methodologies [50,51,52].

### 2.6. Brushing Simulation and Wear Depth Measurements

Discs from the provisional crown material were prepared in a similar manner to the shear bond strength testing methodology. After that, using a graduated transfer pipette, an equal amount of the resin coatings (20 ± 2) mg was dropped on the exposed surface of the provisional crown discs corresponding to the five resin coating groups, and the coated surface was covered with a clear matrix, then light-cured for 60 s.

Brushing simulation was conducted using a toothbrushing machine (MEV 3 LC, Odeme Dental Research, Luzerna, SC, Brazil) fitted with soft-bristle toothbrushes positioned perpendicular to the specimen surfaces. A toothpaste slurry was prepared by mixing a low-abrasion toothpaste (Colgate Total Active Prevention Original; RDA = 70) with deionized water at a 1:3 (*w*/*w*) ratio and continuously stirring; 11 mL was used per reservoir. Half of each specimen surface was masked with water-resistant tape to serve as an unbrushed reference. Brushing was performed under a 2 N load at 70 strokes/min for 10,000 cycles, corresponding to approximately 1 year of clinical brushing. A new toothbrush was used for each specimen, and the slurry was renewed every 5000 cycles.

Wear depth was evaluated using a non-contact optical profilometer (Filmetrics^®^ Profilm3D^®^, Filmetrics, San Diego, CA, USA). The interface between the brushed and unbrushed reference areas was scanned three times for each specimen. Step height between the intact and brushed surfaces was measured using phase-shifting interferometry with the manufacturer’s software (Gen 5 Profilm3D^®^ platform, Filmetrics, San Diego, CA, USA). For each scan, three vertical measurement lines were generated over a 500 μm-wide step. A total of 12 measurements per group were obtained and analyzed [53].

### 2.7. Assessment of the Release of Calcium, Phosphate, and Fluoride Ions

A sodium chloride (NaCl) solution (133 mmol/L) was prepared using deionized water and adjusted to pH 4.0 with 50 mmol/L lactic acid. Three specimens with dimensions of 2 × 2 × 12 mm were immersed in 50 mL of the solution, yielding a specimen-to-solution volume ratio of 2.9 mm^3^/mL, which is comparable to the approximately 3.0 mm^3^/mL reported in earlier studies [16,54].

The release of fluoride (F^−^), calcium (Ca^2+^), and phosphate (PO_4_^3−^) ions was measured at 1, 2, 4, 7, 14, 21, 28, 35, 42, 49, 56, 63, and 70 days. At each time point, 2 mL aliquots were withdrawn and replaced with an equal volume of fresh NaCl solution to maintain a constant total volume.

Calcium and phosphate ion concentrations were determined using a colorimetric assay measured with a microplate reader (SpectraMax^®^ M5, Molecular Probes, San Jose, CA, USA), following previously described protocols and using calibrated standard curves [19]. The calibration curves were constructed using five calcium standards (0.08, 0.16, 0.24, 0.32, and 0.40 mmol/L) and five phosphate standards (0.008, 0.016, 0.024, 0.036, and 0.048 mmol/L).

Fluoride ion release was quantified using a fluoride ion–selective electrode (Orion, Cambridge, MA, USA). A standard calibration curve was generated using fluoride standard solutions. 0.5 mL of each sample was mixed with 0.5 mL of total ionic strength adjustment buffer (TISAB; Fisher Scientific, Pittsburgh, PA, USA) [23].

Ion release was expressed as cumulative concentrations. Throughout the experiment, the pH of the storage solution was monitored and readjusted to pH 4.0 using 50 mmol/L lactic acid as needed.

### 2.8. Statistical Analyses

Statistical analyses were performed using Sigma Plot software (version 16.0, SYSTAT, Chicago, IL, USA). Normality test was done using the Shapiro–Wilk test. One-way analysis of variance (ANOVA, Philadelphia, PA, USA) was used to detect significant differences among groups, followed by Tukey’s post hoc multiple comparison test. Statistical significance was set at a *p*-value < 0.05.

## 3. Results

### 3.1. TEM Analysis of Nanoparticles

**Figure 1:**
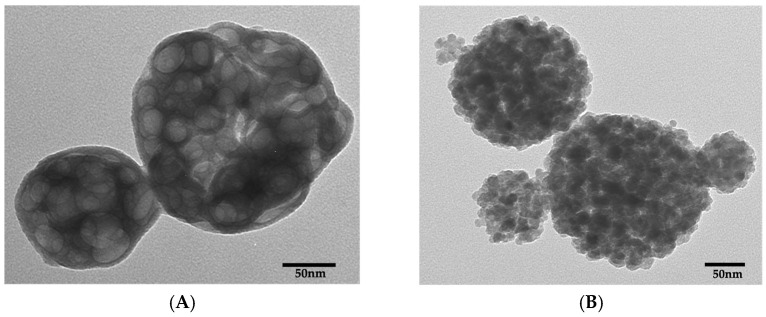
TEM micrographs of nanoparticles of amorphous calcium phosphate (NACP) and calcium fluoride (nCaF_2_) synthesized via a spray-drying technique. (**A**) TEM micrographs of NACP showing clusters that contained spherical particles about 14 nm to 107 nm in diameter; (**B**) TEM micrograph showing an agglomeration of the nCaF_2_, with particle size ranging from 6.60 to 58.97 nm.

Figure 1A shows a representative TEM micrograph of the spray-dried NACP powder. Particle size analysis using ImageJ (mean ± SD; *n* = 50) revealed an average diameter of (107.74 ± 31.65) nm, with a median of 101.78 nm. Morphologically, the particles appeared to consist of aggregates of smaller nanostructures (14–55) nm with multiple spherical surface protrusions, suggesting formation through the coalescence of finer particles during the spray-drying process.

Figure 1B presents a representative TEM image of the calcium fluoride (nCaF_2_) nanoparticles. The particle size ranged from 6.60 to 58.97 nm, with a mean diameter of (27.01 ± 14.38) nm and a median of 25.63 nm (*n* = 50).

### 3.2. Degree of Conversion

**Figure 2:**
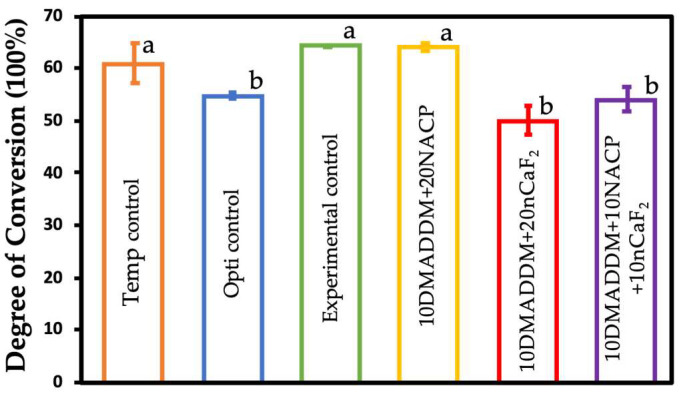
Degree of conversion results of all six groups (mean ± SD; *n* = 3). DC values of all resins tested were above 50%, which was considered sufficient according to the literature. Different letters indicate statistically significant differences among groups (*p* < 0.05).

The degree of conversion results are shown in Figure 2 (mean ± SD; *n* = 3). The DC of the provisional crown material (Temp control) did not differ significantly from that of the experimental control and 10DMADDM+20NACP groups (*p* > 0.05). In contrast, the commercial control (Opti control), 10DMADDM+20nCaF_2_, and 10DMADDM+10NACP+10nCaF_2_ groups demonstrated significantly lower DC values compared with the other groups (*p* < 0.05). Nevertheless, the lowest DC value, approximately 50%, remained within the range considered clinically acceptable according to previous reports [55].

### 3.3. Flow Rate/Flowability

**Figure 3:**
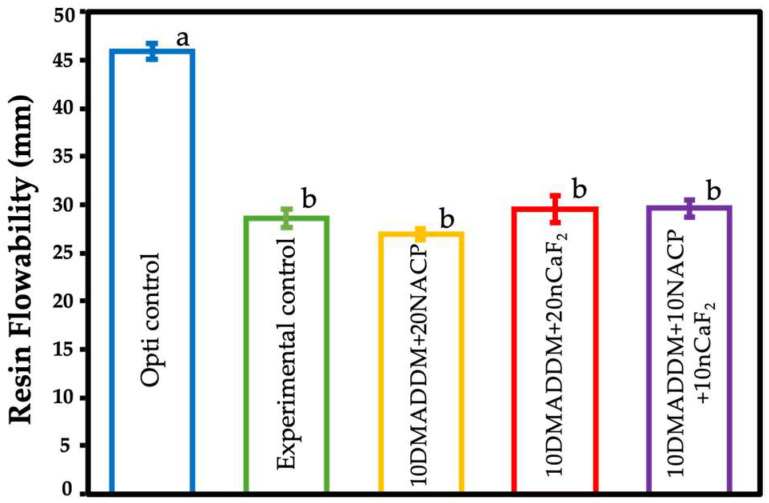
Commercial control, experimental control, and experimental bioactive resin coatings’ flowability were tested (mean ± SD; *n* = 3). Different letters indicate statistically significant differences among groups (*p* < 0.05).

Figure 3 presents the flow properties of the evaluated resin coatings (mean ± SD; *n* = 3). The mean flow values of all groups complied with the ISO requirements, with the exception of the commercial control (Opti control), which exhibited a significantly higher flow rate compared with the experimental formulations (*p* < 0.01). No significant differences were observed among the experimental groups (*p* > 0.05).

### 3.4. Shear Bond Strength

**Figure 4:**
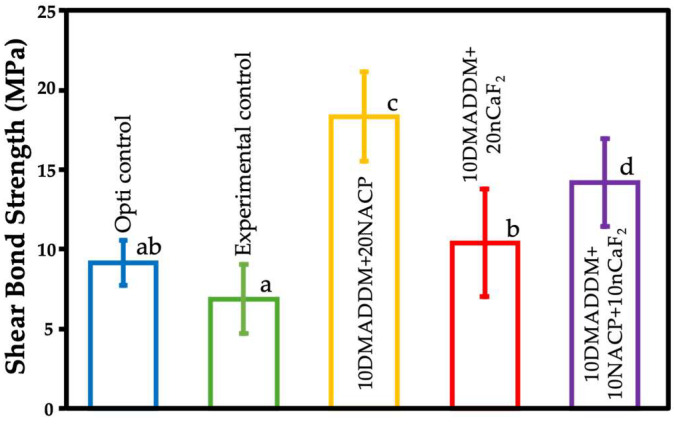
Shear bond strength values of the commercial control, experimental control, and experimental bioactive resin coatings are presented (mean ± SD; *n* = 10). The 10DMADDM+20NACP coating, followed by the 10DMADDM+10NACP+10nCaF2 coating, exhibited the highest bond strength. Different letters indicate statistically significant differences among groups (*p* < 0.05).

The shear bond strength values of the resin coatings to the provisional crown material are presented in Figure 4 (mean ± SD; *n* = 10). The commercial control (Opti control) demonstrated a bond strength of (9.1 ± 1.4 MPa), which was not significantly different from that of the experimental control (6.8 ± 2.1 MPa) and the 10DMADDM+20nCaF_2_ group (10.3 ± 3.3 MPa) (*p* > 0.05). Among the experimental formulations, the 10DMADDM+20NACP group exhibited the highest bond strength (18.3 ± 2.8 MPa), followed by the 10DMADDM+10NACP+10nCaF_2_ group (14.3 ± 2.7 MPa), with a significant difference observed between these two groups (*p* < 0.05). Both 10DMADDM+20NACP and 10DMADDM+10NACP+10nCaF_2_ showed significantly higher bond strength values compared with the remaining groups (*p* < 0.05).

### 3.5. Brushing Wear Depth

**Figure 5:**
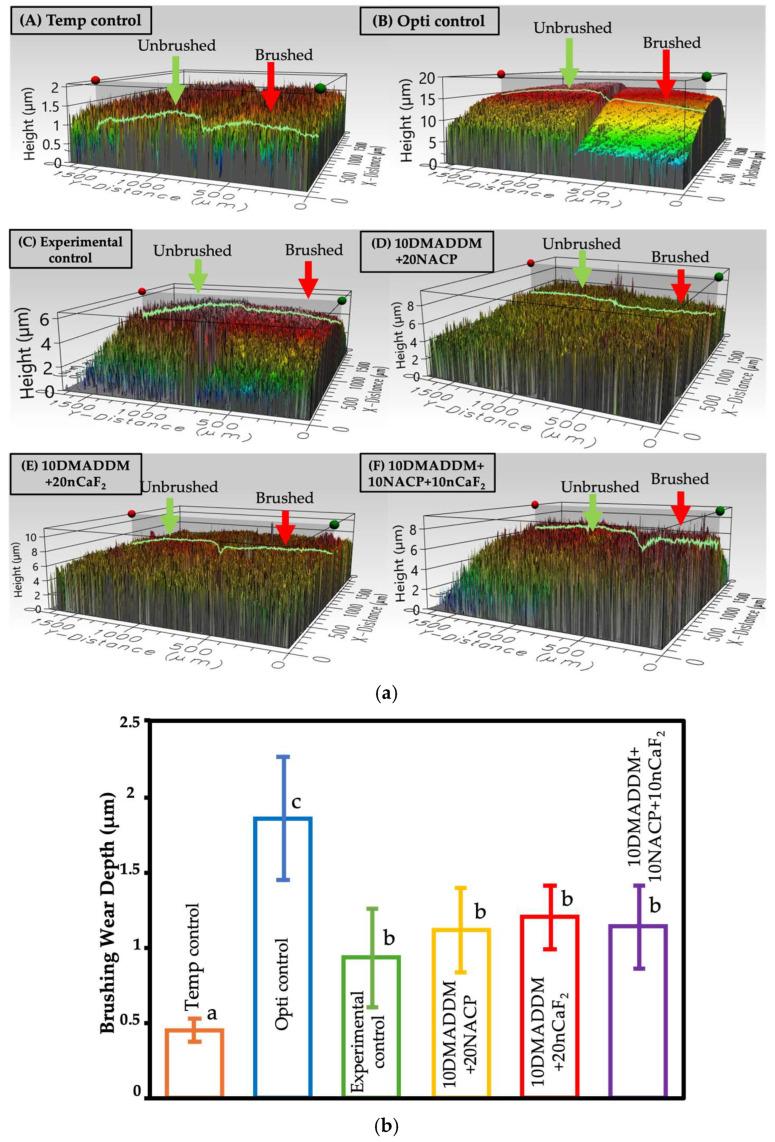
Wear depth results after brushing simulation. (**a**) Representative surface map images from each group showing the step height between the intact and brushed surfaces generated by Filmetrics^®^ Profilm3D^®^ software (Gen 5 Profilm3D^®^ platform, Filmetrics, San Diego, CA, USA), (**b**) Wear depth values (μm) of the provisional crown material and resin coatings following brushing simulation (mean ± SD; *n* = 12). The commercial control Opti control exhibited the highest wear depth, which was significantly greater than that of the other groups (*p* < 0.05). Different letters indicate statistically significant differences among groups (*p* < 0.05).

Figure 5 presents the wear depth of the provisional crown material and resin coatings following brushing simulation (mean ± SD; *n* = 12). The provisional crown material group Temp control showed a wear depth of (0.44 ± 0.07) μm and demonstrated significantly greater wear resistance compared with all other groups (*p* < 0.01). The commercial control Opti control exhibited the highest wear depth (1.85 ± 0.40) μm, which was significantly greater than that of the other groups (*p* < 0.05). The 10DMADDM+20NACP, 10DMADDM+20nCaF_2_, and 10DMADDM+10NACP+10nCaF_2_ groups showed wear depth values of (1.11 ± 0.27, 1.19 ± 0.20, and 1.13 ± 0.27) μm, respectively. These values were not significantly different from that of the experimental control coating (0.93 ± 0.32) μm (*p* > 0.05).

### 3.6. Ions Release for Calcium, Phosphate, and Fluoride

**Figure 6:**
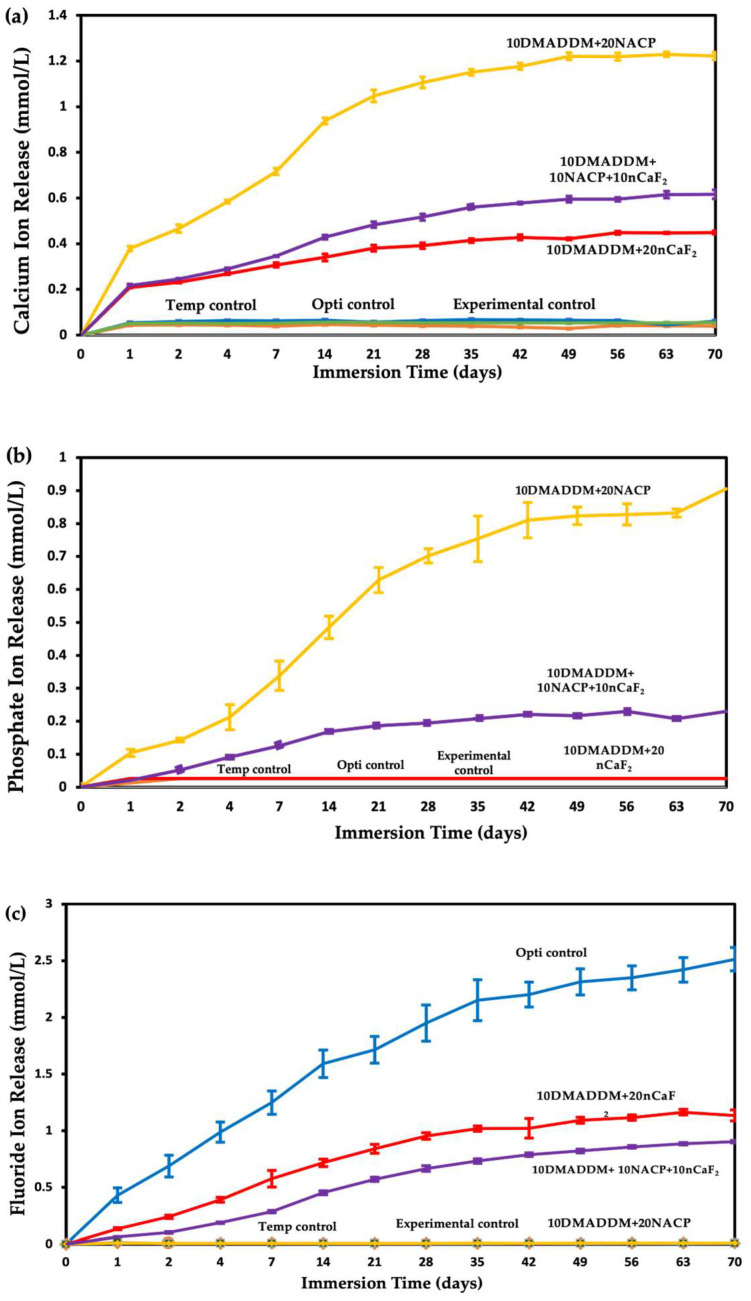
The 70-day release profile of calcium, phosphate, and fluoride ions from provisional crown material, commercial control, and experimental coatings (mean ± SD; *n* = 3). (**a**) Calcium ion release, (**b**) Phosphate ion release, and (**c**) Fluoride ion release.

The release profiles of Ca, PO_4_, and F ions are presented in Figure 6. At 70 days, the resin coating containing 20% NACP (10DMADDM+20NACP) exhibited the highest calcium ion release (1.22 ± 0.01) mmol/L (*p* < 0.05), followed by the coating containing both 10% NACP and 10% nCaF_2_ (10DMADDM+10NACP+10nCaF_2_) (0.62 ± 0.01) mmol/L (*p* < 0.05), and the coating with 20% nCaF_2_ (10DMADDM+20nCaF_2_) (0.45 ± 0.01) mmol/L (*p* < 0.05). The remaining groups showed no detectable calcium ion release.

For phosphate ion release, the 10DMADDM+20NACP group demonstrated the highest level (0.90 ± 0.01) mmol/L (*p* < 0.05), followed by the 10DMADDM+10NACP+10nCaF_2_ group (0.23 ± 0.01) mmol/L (*p* < 0.05). The other groups exhibited negligible phosphate ion release.

Regarding fluoride ion release, the commercial control (Opti control) showed the greatest fluoride release (0.91 ± 0.01) mmol/L (*p* < 0.05), followed by the 10DMADDM+20nCaF_2_ coating (0.41 ± 0.01) mmol/L (*p* < 0.05) and the 10DMADDM+10NACP+10nCaF_2_ coating (0.33 ± 0.02) mmol/L (*p* < 0.05). The remaining groups demonstrated minimal fluoride release, with values below 0.01 mmol/L.

**Figure 7:**
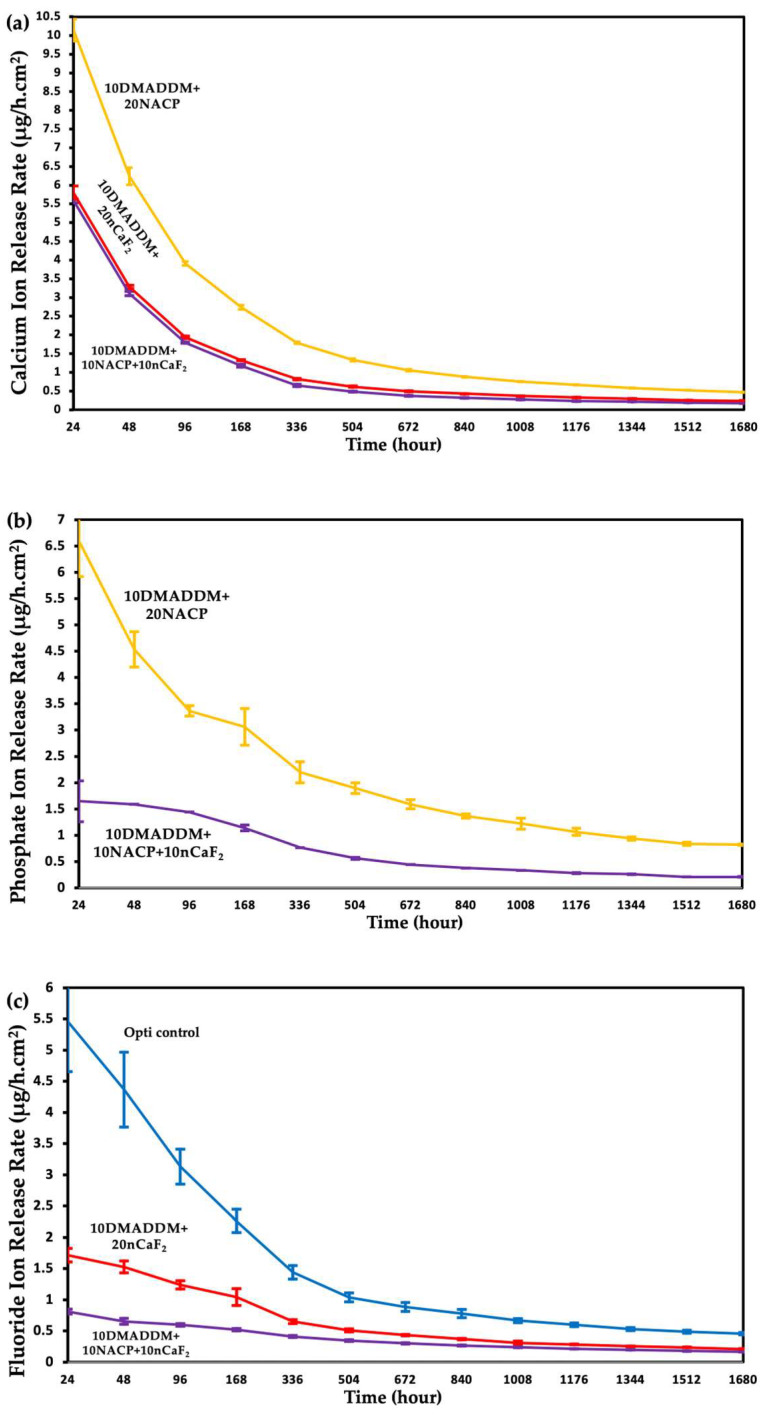
Rate of release for calcium, phosphate, and fluoride ions per hour per specimen surface area from commercial control and experimental coatings (mean ± SD; *n* = 3). Groups with negligible release were not included. (**a**) Calcium ion release rate, (**b**) Phosphate ion release rate, and (**c**) Fluoride ion release rate.

The Ca, PO_4,_ and F profiles of release rate per hour per specimen surface area were calculated from the data in Figure 6 and plotted in Figure 7. Groups with negligible release were not included. The initial Ca release rate for the 10DMADDM+20NACP group reached 10.13 μg/(h·cm^2^), and the rate decreases with the steepest drops to 6.24 μg/(h·cm^2^) after 48 h and to 3.91 μg/(h·cm^2^) after 96 h, and gradually declines to a plateau. The same pattern of release was explored for 10DMADDM+20nCaF_2_ and 10DMADDM+10NACP+10nCaF_2_ groups with initial Ca release rates of 5.58 μg/(h·cm^2^) and 5.80 μg/(h·cm^2^) respectively, followed by a sharp drop after 96 h to 1.79 μg/(h·cm^2^) and 1.94 μg/(h·cm^2^). Both groups plateau in concentration but the rate keeps dropping as time progresses.

The initial PO_4_ release rate of the 10DMADDM+20NACP group reached 6.60 μg/(h·cm^2^); it decreased sharply to 4.53 μg/(h·cm^2^) after 48 h and gradually declined as time progressed to a plateau. The initial release rate of the 10DMADDM+10NACP+10nCaF_2_ group reached 1.65 μg/(h·cm^2^) with a declining rate pattern as time progressed until it plateaued in concentration.

The F release rate of the Opti control group initially reached 5.46 μg/(h·cm^2^) and dropped to 1.44 μg/(h·cm^2^) after 300 h and gradually declined to a plateau. The initial release rate of the 10DMADDM+20nCaF_2_ group reached 1.72 μg/(h·cm^2^) and gradually declined as time progressed to a plateau. The same pattern was explored with 10DMADDM+10NACP+10nCaF_2_, as the initial rate reached 0.81 μg/(h·cm^2^) with a declining release rate as time progressed until it plateaued.

## 4. Discussion

The present study developed a multifunctional resin-based coating incorporating antibacterial and remineralizing components within a UDMA/TEG-DVBE polymer system. The results demonstrated that the experimental coatings achieved an acceptable degree of conversion, flow behavior, bonding performance, wear resistance, and sustained ion release. These results support the proposed hypotheses and highlight their potential for clinical application in provisional restorations.

The incorporation of specifically 10% DMADDM into the experimental formulations was based on our previous publication, in which this concentration demonstrated the highest antibacterial efficacy against *S. mutans* biofilms within the same UDMA/TEG-DVBE resin system, while maintaining mechanical integrity and good surface characteristics [42]. Earlier studies reported that lower concentrations may result in insufficient antibacterial activity, whereas higher concentrations could adversely affect polymerization and mechanical properties [56]. Therefore, the present formulation aimed to balance antibacterial effectiveness and physicochemical stability. The degree of conversion observed in all experimental groups indicates that the addition of DMADDM did not significantly impair the polymerization process, which is consistent with prior findings showing that DMADDM can be covalently incorporated into the polymer network without compromising structural integrity [57].

The degree of conversion significantly influences the mechanical properties, durability, and biocompatibility of resin-based materials. In the present study, although a reduction in DC was observed in some groups, the lowest value remained approximately 50%, which is considered clinically acceptable according to previous reports [55]. This threshold has been associated with sufficient network formation to ensure adequate mechanical performance and reduced leaching of unreacted monomers. The slightly lower DC observed in formulations containing both NACP and nCaF_2_ may be attributed to increased light scattering and reduced light transmission caused by the presence of nanoparticles, which has also been reported in previous studies involving bioactive fillers [36,58,59].

The clinical handling and adaptation of resin coatings play a major role in clinicians’ decisions on whether to use the material or not. Therefore, flow characteristics are a very important factor in determining clinical operability. The 20% NACP and nCaF_2_ filler concentrations were selected based on preliminary flowability assessments to ensure acceptable handling properties for surface coating applications, and are consistent with prior studies demonstrating sustained ion release at this filler level. All experimental groups complied with ISO requirements, indicating adequate viscosity and clinical applicability. The significantly higher flow observed in the commercial control may be due to its unfilled resin composition, and these results are consistent with previous reports demonstrating that filler incorporation increases viscosity and reduces flow [60]. The controlled flow behavior of the experimental coatings is advantageous for maintaining uniform coating thickness and improving marginal adaptation in provisional restorations. Furthermore, the light-cured nature of the formulation provides extended working time for precise chairside placement, and the thin coating thickness previously characterized for this system is unlikely to interfere with occlusal relationships or marginal adaptation. Taken together, these handling characteristics support the clinical translatability of the developed coatings and their compatibility with routine chairside provisional crown workflows.

The bonding strength of the resin-based coating applied to provisional materials is very important. The present study demonstrated that all experimental coatings achieved bond strengths exceeding 5 MPa, which is the minimum requirement as specified by ISO 10477 for polymer-based materials [61]. Notably, the NACP-containing formulations exhibited significantly higher bond strength compared with other groups. This improvement may be attributed to enhanced interfacial interactions and possible chemical bonding between calcium phosphate particles and the resin matrix, which has been reported in previous studies [62]. Furthermore, the flexible and hydrolytically stable UDMA/TEG-DVBE matrix may have contributed to improved stress distribution and interfacial stability.

Shear bond strength appeared to be correlated with the degree of conversion. Higher DC values indicate a greater extent of monomer polymerization and crosslink density, resulting in improved mechanical integrity and interfacial cohesion. A well-polymerized network enhances stress distribution at the bonded interface and reduces the presence of residual unreacted monomers, which could otherwise weaken the adhesive joint. Conversely, reduced DC may lead to less network formation, lower mechanical strength, and compromised interfacial stability. This relationship likely explains the observed trend between DC and shear bond strength among the tested groups [26].

Wear resistance is another critical factor influencing the long-term performance of resin-based coatings. The brushing simulation results indicate that the experimental coatings exhibited comparable wear to the experimental control and significantly improved wear resistance compared with the commercial control. These findings are consistent with previous studies showing that nanofillers can enhance resistance to abrasion by reinforcing the polymer network and reducing matrix degradation [63,64]. The relatively low wear depth of the multifunctional coatings suggests their suitability for long-term intraoral use.

Sustained ion release from the experimental coatings confirmed the successful incorporation of bioactive nanoparticles. The NACP-containing groups demonstrated prolonged calcium and phosphate ion release, which is essential for remineralization and buffering acidic environments. The presence of nCaF_2_ contributed to fluoride release, which is known to enhance remineralization, inhibit demineralization, and reduce bacterial acid production [30,65]. The combined NACP–nCaF_2_ formulation exhibited dual ion release, supporting the synergistic approach to caries prevention. Previous studies have reported that such multifunctional systems can significantly improve resistance to secondary caries by promoting mineral deposition and reducing acidogenic bacterial activity [24,25].

The release rate profiles shown in Figure 7 provide important insight into the kinetics of ion release from the experimental coatings and allow direct comparison with previously reported values for clinically used materials. The release rate behavior of NACP is governed by several factors. The NACP particles, with a mean diameter of 107.74 nm and high specific surface area, serve as the primary calcium and phosphate ion source, with their content directly determining the quantity of available ions [23]. The interfaces between NACP particles and the resin matrix act as pathways that facilitate water diffusion inward and ion diffusion outward, enhancing release beyond what would be expected from filler content alone [19]. Importantly, NACP exhibits pH-responsive behavior as it greatly increases ion release under acidic conditions, with previous studies demonstrating a 5–10 fold increase as pH decreased from neutral to a cariogenic pH of 4, making NACP a “smart” material that releases ions most when they are needed to combat demineralization [19,26]. In the present study, specimens were immersed at pH 4, which maximized the NACP ion release contribution, consistent with the high initial calcium release rate of 10.13 µg/(h·cm^2^) observed for the 10DMADDM+20NACP group, which declined to 0.47 µg/(h·cm^2^) at 70 days. This pattern is consistent with diffusion-controlled release from bioactive nanoparticle systems, reflecting progressive depletion of surface-accessible ions characteristic of such materials [23].

The chemical composition of the two nanoparticles explains the observed differences in calcium ion release between groups. NACP, with a formula of Ca_3_(PO_4_)_2_, contains three calcium ions per molecule, whereas nCaF_2_ contains only one. Therefore, when the total filler is divided equally between NACP and nCaF_2_ in the dual formulation, the available calcium is substantially lower than in the 20% NACP group alone, directly explaining why the 10DMADDM+10NACP+10nCaF_2_ group released less calcium (0.62 mmol/L) than the 10DMADDM+20NACP group (1.22 mmol/L) at 70 days. This is not a limitation of the dual formulation, as the reduction in calcium release is intentionally offset by the introduction of sustained fluoride release, which the 20% NACP group alone cannot provide. Particle size further influenced release kinetics differently for each filler. TEM analysis showed that nCaF_2_ particles were substantially smaller (mean 27 nm) than NACP particles (mean 108 nm). Smaller particles have greater surface area, which promotes faster ion dissolution and likely explains the higher initial fluoride release rate in nCaF_2_-containing groups, consistent with previously reported particle size–release relationships [23]. The initial fluoride release rate of the 10DMADDM+20nCaF_2_ group reached 1.72 µg/(h·cm^2^), exceeding the initial release rates reported for Fuji II glass ionomer (~0.4 µg/(h·cm^2^)) and Vitremer resin-modified glass ionomer (~0.4 µg/(h·cm^2^)), and was comparable to the 1.6 µg/(h·cm^2^) reported for a resin filled with 23% commercial CaF_2_ at pH 4 [66]. The dual 10DMADDM+10NACP+10nCaF_2_ formulation demonstrated an initial fluoride release rate of 0.81 µg/(h·cm^2^), which remained above the sustained release rates of 0.03–0.1 µg/(h·cm^2^) reported for conventional glass ionomer and resin-modified glass ionomer materials at 50 days [67]. At 70 days (1680 h), both nCaF_2_-containing formulations maintained fluoride release rates substantially exceeding the ~0.05 µg/(h·cm^2^) reported at 70 days for a resin containing 23% commercial CaF_2_ powder [66,67].

In the dual 10DMADDM+10NACP+10nCaF_2_ formulation, NACP plays a mediating role in the overall release rate profile. The calcium released from NACP does not simply add to the total ion pool; it actively participates in the remineralization reaction by providing the calcium ions that fluoride alone cannot supply. Despite the declining release rate over time, ion release was maintained continuously over 70 days, which is clinically relevant as sustained ion availability, even at lower rates, is sufficient to maintain local calcium, phosphate, and fluoride concentrations above the critical thresholds required for remineralization. The rationale for the dual formulation is therefore synergistic rather than simply additive. Fluorapatite formation requires calcium, phosphate, and fluoride ions simultaneously. By combining NACP and nCaF_2_, the dual formulation ensures their synchronized availability, enabling fluorapatite formation that neither component alone at equivalent filler levels could achieve. Taken together, the fluoride release rates of the nCaF_2_-containing experimental coatings are comparable to or exceed those of commercial glass ionomer materials already used clinically for their fluoride-releasing properties, supporting the clinical potential of the developed coatings as therapeutic surface treatments for provisional restorations.

The UDMA/TEG-DVBE resin matrix offered several advantages over conventional methacrylate systems. The reduced ester content and enhanced hydrolytic stability associated with ether-based monomers may improve resistance to biodegradation and enzymatic attack in the oral environment [39]. This characteristic is important for provisional restorations, which are exposed to saliva, enzymes, and bacterial metabolites for extended periods. Moreover, the improved network homogeneity and reduced polymerization stress associated with this system may contribute to long-term bonding stability and reduced marginal degradation [25].

From a clinical perspective, the multifunctional resin-based coating developed in this study could provide an effective approach for enhancing the longevity of provisional restorations. By combining antibacterial and remineralizing functions, these coatings may reduce biofilm formation, prevent demineralization, and improve wear resistance. The surface coating approach also offers versatility and ease of application, allowing clinicians to upgrade existing materials without altering their bulk properties.

The optical properties and color stability of the experimental coatings were not evaluated in the present study. Future investigations should incorporate spectrophotometric assessment of color change (ΔE), translucency, and gloss to provide a comprehensive characterization of the esthetic performance of the developed formulations.

FTIR characterization of the formulated coatings was not performed in the present study. Future investigations should incorporate FTIR analysis to provide direct instrumental evidence of chemical interactions between the polymer matrix and the surface functional groups of the incorporated nanoparticles, and to further characterize the degree of conversion across experimental formulations. Also, microscopic analysis, including SEM/TEM examination of nanoparticle distribution within the polymer matrix, bonded interface characterization, and systematic failure mode classification, was not performed in the present study and represents an important direction for future investigation.

Future studies should further investigate the long-term antibacterial efficacy of these coatings using clinically relevant biofilm models. A multispecies and human saliva-derived microcosm biofilm system should be evaluated before and after mechanical wear to simulate oral conditions more accurately. In addition, surface roughness and wettability changes following brushing should be assessed, as these factors influence bacterial adhesion and biofilm formation. Contact angle measurements could provide insight into surface energy and its role in bacterial colonization. Furthermore, long-term aging, thermal cycling, and enzymatic degradation studies are required to validate the durability and clinical applicability of these coatings.

Within the limitations of this in vitro study, the multifunctional resin coatings demonstrated promising antibacterial and remineralizing potential while maintaining acceptable physicochemical and mechanical properties. These findings support the continued development of therapeutic polymer coatings for preventive and restorative dental applications.

## 5. Conclusions

The multifunctional UDMA/TEG-DVBE-based resin coating successfully integrated antibacterial (10% DMADDM) and remineralizing nanoparticles without compromising physicochemical performance. All groups maintained clinically acceptable polymerization, with DC values ≥50%. Incorporation of 20% NACP increased shear bond strength up to 18.3 MPa; approximately 2.7-fold higher than the experimental control and well above the ISO 10477 threshold (5 MPa). Brushing simulation (10,000 cycles) demonstrated surface loss below 1.2 µm for experimental coatings, reducing wear by approximately 40% compared with the commercial coating. Sustained ion release was maintained for 70 days, reaching 1.22 mmol/L calcium and 0.90 mmol/L phosphate, while the dual formulation achieved calcium and fluoride release. These findings demonstrate that the developed coatings provide mechanically stable, wear-resistant, and therapeutically active surfaces, indicating strong potential for improving the longevity of provisional crowns and restorations. Despite the promising findings, this study has several limitations, including the absence of long-term aging protocols, optical property evaluation, direct nanoparticle distribution characterization, and microscopic interfacial analysis. Future studies should investigate antibacterial efficacy by evaluating multi-species biofilm performance, incorporating thermocycling, water storage aging, SEM/TEM nanoparticle analysis, and optical characterization to provide a more comprehensive evaluation of the developed coatings.

## Data Availability

The raw data supporting the conclusions of this article will be made available by the authors on request.

## Abbreviations

The following abbreviations are used in this manuscript:

DMADDM	Dimethylaminododecyl Methacrylate
UDMA	Urethane Dimethacrylate
Bis-GMA	Bisphenol A Glycidyl Methacrylate
QAM	Quaternary Ammonium Methacrylate
TEG-DVBE	Triethylene Glycol Divinylbenzyl Ether
NACP	Nanoparticles of Amorphous Calcium Phosphate
nCaF_2_	Calcium Fluoride Nanoparticles
ISO	International Organization for Standardization
RDA	Relative Dentin Abrasivity
TEM	Transmission Electron Microscopy
SD	Standard Deviation
MPa	Megapascal
DC	Degree of Conversion
